# Emergence of resistance to novel β-lactam/β-lactamase inhibitor combinations in KPC-producing *Klebsiella pneumoniae*: clinical and genomic insights from consecutive bloodstream infections

**DOI:** 10.1007/s15010-025-02716-4

**Published:** 2025-12-23

**Authors:** Matteo Boattini, Sara Comini, Guido Ricciardelli, Lisa Pastrone, Roberto Casale, Luisa Guarrasi, Rossana Cavallo, Cristina Costa, Paolo Gaibani, Gabriele Bianco

**Affiliations:** 1 Department of Public Health and Paediatrics, University of Torino, Turin, Italy; 2 Microbiology and Virology Unit, University Hospital Città Della Salute E Della Scienza Di Torino, Turin, Italy; 3 Lisbon Academic Medical Centre, Lisbon, Portugal; 4 Operative Unit of Clinical Pathology, Carlo Urbani Hospital, Ancona, Italy; 5 Microbiology and Virology Unit, Department of Pathology, Azienda Ospedaliera Universitaria Integrata Di Verona, Verona, Italy; 6 Department of Diagnostic and Public Health, Microbiology Section, University of Verona, Verona, Italy; 7 Department of Experimental Medicine, University of Salento, Via Provinciale Monteroni N. 165, 73100 Lecce, Italy; 8 Microbiology and Virology Unit, Vito Fazzi Hospital, Lecce, Italy

**Keywords:** Aztreonam/avibactam, Ceftazidime/avibactam, KPC variant, Imipenem/relebactam, Meropenem/vaborbactam, Novel β-lactam/β-lactamase inhibitor

## Abstract

**Purpose:**

Novel β-lactam/β-lactamase inhibitor combinations (BL/BLICs) such as ceftazidime/avibactam (CAZ/AVI), meropenem/vaborbactam (MEM/VAB), imipenem/relebactam (IMP/REL) and aztreonam/avibactam (ATM/AVI) have expanded therapeutic choices against KPC-producing *K. pneumoniae*. However, emerging resistance threatens their long-term efficacy. We investigated the prevalence, genomic mechanisms, and clinical correlates of resistance to these agents among KPC-producing *K. pneumoniae* bloodstream isolates.

**Methods:**

Consecutive KPC-producing *K. pneumoniae* bloodstream isolates collected between 2021 and 2024 at a tertiary university hospital were tested for susceptibility to novel BL/BLICs and comparators. Whole-genome sequencing (WGS) was performed on isolates resistant to any BL/BLIC to characterise genetic backgrounds. Clinical data from corresponding patients were analysed to explore risk factors and outcomes.

**Results:**

Among 178 K*. pneumoniae* isolates, ATM/AVI, IMP/REL and MEM/VAB retained excellent in vitro activity (≥ 96% susceptible), while 11% were resistant to CAZ/AVI. One hundred fifty-four (86.5%) were susceptible to all BL/BLICs, whereas 24 (13.5%) displayed resistance to at least one agent, most commonly CAZ/AVI. WGS revealed a genetically diverse population mainly comprising high-risk clones ST512 and ST101. Resistance was driven by KPC variants (KPC-31, KPC-167, KPC-93, KPC-49, KPC-14, KPC-121, KPC-33) and porin disruptions (OmpK36 insertions, OmpK35 loss). Most patients (91%) had prior colonisation and recent β-lactam exposure; median time to resistance emergence was 47 days. The 28-day mortality among patients with BL/BLIC-resistant infections was 21.7%.

**Conclusion:**

Resistance to novel BL/BLICs among KPC-producing *K. pneumoniae* is emerging in Italian hospitals, largely mediated by *bla*_KPC_ variants and porin defects under selective antibiotic pressure. While ATM/AVI, MEM/VAB and IMP/REL remain highly active, resistance to CAZ/AVI is increasingly frequent. Continuous genomic surveillance and optimised antimicrobial stewardship are essential to preserve the efficacy of these last-line agents.

## Introduction

Antimicrobial resistance represents one of the most critical global public health challenges, contributing to increasing morbidity, mortality, and healthcare expenditures worldwide [1]. Among multidrug-resistant pathogens, carbapenemase-producing Enterobacterales (CPE) are of particular concern because of their limited therapeutic options and strong association with adverse clinical outcomes [2]. Within this group, *Klebsiella pneumoniae* has emerged as a predominant threat, largely due to the production of *K. pneumoniae carbapenemases* (KPCs)—enzymes capable of hydrolysing nearly all β-lactam antibiotics, including carbapenems [3, 4]. KPC enzymes belong to Ambler class A β-lactamases and are poorly inhibited by traditional β-lactamase inhibitors such as clavulanic acid, sulbactam, and tazobactam [5]. The introduction of novel β-lactam/β-lactamase inhibitor combinations (BL/BLICs) has markedly expanded the therapeutic armamentarium against KPC-producing *K. pneumoniae* (KPC-Kp). Agents as ceftazidime/avibactam (CAZ/AVI), meropenem/vaborbactam (MEM/VAB), imipenem/relebactam (IMP/REL), and aztreonam/avibactam (ATM/AVI) have demonstrated potent in vitro and substantial clinical efficacy against KPC-producing strains [6–10].Resistance to these novel agents has nonetheless emerged rapidly across multiple countries following their clinical introduction [6, 11, 12]. Reported mechanisms include mutations within *bla*_KPC_ genes, porin loss, β-lactamase overexpression, and altered penicillin-binding proteins, often acting synergistically to confer resistance [6, 11–13]. The widespread use of CAZ/AVI, the first of these combinations to achieve broad clinical adoption, has imposed substantial selective pressure, promoting the spread of KPC variants with reduced avibactam susceptibility or enhanced hydrolytic activity against ceftazidime [11, 13]. CAZ/AVI-resistant KPC-Kp isolates are most frequently observed in patients experiencing prolonged or suboptimal β-lactam exposure [12]. Moreover, clinical factors such as critical illness, obesity, renal dysfunction, and respiratory tract infections have been associated with inadequate pharmacokinetic target attainment, potentially facilitating the selection of resistant subpopulations [9, 14, 15].Although MEM/VAB has retained robust activity against most KPC-producing strains, sporadic resistance has been reported. These cases are predominantly associated with reduced outer membrane permeability due to loss or modification of the OmpK35 and OmpK36 porins, frequently coupled with KPC overexpression [6, 11]. Such resistant isolates are typically recovered from patients with prior exposure to MEM/VAB or CAZ/AVI, underscoring the role of sequential BL/BLIC exposure in selecting multidrug-resistant subpopulations [11, 12].

Although uncommon, IMP/REL resistance has been documented in isolates exhibiting decreased porin expression or structural alterations in combination with high-level carbapenemase production [6, 11].

Approved in 2024, ATM/AVI showed potent in vitro activity against both KPC- and metallo-β-lactamase-producing Enterobacterales, although data on resistance mechanisms and associated infections remain limited [16–18].Infections caused by KPC-producing strains resistant to novel BL/BLICs are associated with delayed initiation of effective therapy and increased mortality compared with infections due to susceptible isolates [9, 14, 16, 17]. Italy remains among the European countries with the highest endemicity of KPC-Kp [19]. Although the introduction of new BL/BLICs since 2018 has substantially improved clinical outcomes, the emergence of resistance poses a serious threat to their long-term efficacy. Several Italian studies have evaluated the in vitro activity of these agents against local isolates; however, comprehensive data integrating resistance mechanisms, prior antimicrobial exposure, and the clinical characteristics and outcomes of infections caused by resistant isolates remain scarce [6, 10, 12].This study aimed to assess the in vitro activity of recently approved BL/BLICs (CAZ/AVI, MEM/VAB, IMP/REL, and AZT/AVI) alongside selected comparators, against consecutive KPC-producing Enterobacterales isolates collected at a university hospital in Northern Italy between 2021 and March 2024. In addition, the study sought to characterize the clinical features and outcomes of patients with infections caused by isolates resistant to novel BL/BLICs, and to elucidate the underlying molecular mechanism of resistance through whole-genome sequencing (WGS).

In addition, the study sought to characterize the clinical features and outcomes of patients with infections caused by isolates resistant to these novel BL/BLICs, and to elucidate the underlying molecular mechanisms of resistance through whole-genome sequencing (WGS).

## Material and methods

### Study design

This prospective observational study was conducted at the University Hospital Città della Salute e della Scienza di Torino (Turin, Italy). Consecutive KPC-Kp isolates obtained from bloodstream infections in hospitalized patients admitted between January 1, 2021, and March 31, 2024, were included. In addition to routine diagnostic procedures—including species identification, in vitro antimicrobial susceptibility testing, and phenotypic and/or genotypic confirmation of KPC production—all isolates were tested against an extended antibiotic panel comprising four novel BL/BLICs: CAZ/AVI, MEM/VAB, IMP/REL, ATM/AVI.

Isolates classified as resistant to novel BL/BLICs according to EUCAST criteria underwent WGS analysis. Concurrently, the corresponding patient electronic medical records were reviewed to obtain the following data: demographic information, comorbidities, prior antibiotic exposure, infection characteristics, and clinical outcomes.

### Blood culture workflow and isolate collection

Blood culture processing was performed as previously described [20]. Briefly, BACT/ALERT FA and FN Plus blood culture bottles (bioMérieux, Marcy l’Étoile, France) were incubated in the BACT/ALERT Virtuo system (bioMérieux). Positive bottles were subjected to Gram staining and subcultured onto appropriate solid media. Microbial identification was carried out from overnight subcultures using matrix-assisted laser desorption ionization–time of flight mass spectrometry (MALDI-TOF MS, Bruker Daltonik GmbH, Bremen, Germany). Antimicrobial susceptibility testing was performed on overnight subcultures using the Microscan WalkAway Plus system according to the manufacturer’s instructions (Beckman Coulter, Brea, CA, USA). Carbapenemase production in Enterobacterales was assessed by the genotypic assay Xpert Carba-R on the GeneXpert platform (Cepheid, Sunnyvale, CA, USA) when meropenem MIC exceeded 0.12 mg/L.All *K. pneumoniae* isolates identified as positive for KPC production or carrying *bla*_KPC_ during the study period were collected and cryopreserved in 50% glycerol/50% cation-adjusted Mueller–Hinton broth (CAMHB) at − 80 °C for subsequent analyses. Duplicate isolates from the same patient exhibitibg identical antimicrobial susceptibility testing profiles were considered part of a single bloodstream infection episode and were excluded from further analysis.

### Antimicrobial susceptibility testing

Following thawing, isolates were subcultured twice on blood agar to ensure viability, and species identification was confirmed using MALDI-TOF MS. Antimicrobial susceptibility testing was carried out using the Sensititre^™^ broth microdilution system (EUMDROXF panel, Thermo Scientific) for the following agents: CAZ/AVI (0.25–16 mg/L), MEM/VAB (0.06–16 mg/L), IMP/REL (0.06–8 mg/L), ceftolozane/tazobactam (0.25–8 mg/L), piperacillin/tazobactam (4–32 mg/L), cefepime (1–16 mg/L), meropenem (0.12–16 mg/L), imipenem (1–8 mg/L), aztreonam (1–32 mg/L), tigecycline (0.5–1 mg/L), eravacycline (0.008–0.5 mg/L), amikacin (2–32 mg/L), tobramycin (0.5–4 mg/L) and colistin (0.5–16 mg/L). Susceptibility to ATM/AVI was assessed separately by broth microdilution (0.25–64 mg/L). MIC values were interpreted according to EUCAST version 15.0 [21] and CLSI M100 ED35:2025 breakpoints [22].

### Whole genome sequencing analysis

WGS of KPC-Kp strains resistant to CAZ/AVI and/or MEM/VAB and/or IMP/REL was performed as previously described [6].

Briefly, libraries were generated from 300 ng genomic DNA using from an overnight culture on Mueller–Hinton agar plates using PureLink^®^ Genomic DNA Purification Kit (Invitrogen, USA) following the manufacturer’s instructions. Libraries were prepared with DNA Prep Library Preparation Kit with iSeq Reagent Kit v.2 using 2 × 150 paired-end reads and sequenced by llumina iSeq100 platform (Illumina, USA) with 2 × 300 paired-end MiSeq kit V3 with a median coverage, × 30. Prior to genome assembly, a filtering step was conducted to remove any contaminant human reads. Briefly, a read quality evaluation was performed with FastQC 0.11.9 and aggregated MultiQC reporting before and after filtering. Adapter and quality trimming (Trimmomatic) was applied, and, where applicable, host-derived reads were removed by mapping to the host reference genome (e.g., GRCh38) using BWA-MEM/BBMap and retaining only unmapped reads. A minimum Q score of 30 was set to filter Illumina reads, Filtered sequencing reads were de novo assembled using SPAdes software v3.15.2.

Multi-locus sequence type and serotype analysis were performed with MLST v2.23.0 (https://github.com/tseemann/mlst) and Kaptive v2.0.6 (https://github.com/klebgenomics/Kaptive), respectively. Antimicrobial resistance genes were determined by using Abricate v1.0.1 (https://github.com/tseemann/abricate) and AMRFinderPlus v3.11.14 (https://github.com/ncbi/amr), and results were manually inspected by BLAST v2.14.0 (https://blast.ncbi.nlm.nih.gov/Blast.cgi).

### Clinical data collection

For each patient with a bloodstream infection caused by isolates resistant to the novel BL/BLICs identified during the study period, detailed clinical data were collected. These included demographic characteristics, comorbidities, reasons for hospital admission, documented prior colonization with KPC-Kp, and prior exposure to novel BL/BLICs. Information on admistered antibiotic regimens, time to emergence of resistance, targeted therapy, sepsis-associated complications, duration of hospitalization, and clinical outcomes was also recorded.

These included demographic characteristics, comorbidities, reasons for hospital admission, documented prior colonization with KPC-producing *Klebsiella pneumoniae* (KPC-Kp), and previous exposure to novel BL/BLICs. Information on administered antibiotic regimens, time to resistance emergence, targeted therapy, sepsis-associated complications, duration of hospitalization, and clinical outcomes was also recorded.

## Results

Over the study period, a total of 178 consecutive KPC-Kp isolates were identified (Table 1). AZT/AVI exhibited the highest in vitro activity, with 99.4% of isolates classified as susceptible (MIC₅₀ ≤ 0.25 mg/L; MIC₉₀ 2 mg/L), followed by IMP/REL (97.7% susceptible by EUCAST and 89.3% susceptible by CLSI; MIC_50_ = 0.25 mg/L; MIC_90_ = 2 mg/L) and MEM/VAB (96.6% susceptible by EUCAST and 93.2% susceptible by CLSI; MIC_50_ = 0.25 mg/L; MIC_90_ = 4 mg/L). In contrast, CAZ/AVI showed reduced activity, with 11.2% of isolates classified as resistant (MIC_50_ = 2 mg/L and MIC_90_ > 16 mg/L (Table 1).

**Table 1:** In vitro activity of aztreonam/avibactam, ceftazidime/avibactam, meropenem/vaborbactam, imipenem/relebactam, and comparators against the collection of KPC-Kp bloodstream infections isolates (n = 178)

	MIC range mg/L	MIC_50_ mg/L	MIC_90_ mg/L	EUCAST breakpoints			CLSI breakpoints			
				Susceptible (%)	Susceptible increased exposure (%)	Resistant (%)	Susceptible (%)	Susceptible-dose dependent (%)	Intermediate (%)	Resistant (%)
AZT/AVI	≤ 0.25 to 16	≤ 0.25	2	176 (99.4)	–	1 (0.6)	NA	NA	NA	NA
CAZ/AVI	≤ 0.25 to > 16	2	> 16	158 (88.8)	–	20 (11.2)	158 (88.8)	–	–	20 (11.2)
MEM/VAB	≤ 0.06 to > 16	0.25	4	172 (96.6)	–	6 (3.4)	166 (93.2)	–	6 (3.4)	6 (3.4)
IMP/REL	≤ 0.06 to > 8	0.25	2	174 (97.7)	–	4 (2.2)	159 (89.3)	–	15 (8.5)	4 (2.2)
MEM	≤ 0.12 to > 16	> 16	8	7 (3.9)	9 (5.1)	107 (91)	0	–	7 (3.9)	171 (96.1)
IMP	≤ 1 to > 8	> 8	4	12 (6.8)	4 (2.2)	162 (91)	11 (6.2)	–	1 (0.6)	166 (93.2)
C/T	4 to > 8	> 8	> 8	0	–	178 (100)	0	–	1 (0.6)	177 (99.4)
PIP/TAZ	16 to > 32	> 32	> 32	1 (0.6)	–	177 (99.4)	0	1 (0.6)	–	177 (99.4)
AZT	2 to > 32	> 32	> 32	0	2 (1.1)	176 (98.9)	2 (1.1)	–	1 (0.6)	177 (99.4)
FEP	8 to > 16	> 16	> 16	0	1 (0.6)	177 (99.4)	0	1 (0.6)	0	177 (99.4)
AK	≤ 2 to > 32	32	> 32	58 (32.6)	–	120 (67.4)	39 (21.9)	–	5 (2.8)	134 (75.3)
COL	≤ 0.5 to > 16	≤ 0.5	> 16	145 (81.5)	–	33 (18.5)	-	–	145 (81.5)	33 (18.5)
TOB^a^	≤ 0.5 to > 4	> 4	> 4	48 (27)	–	130 (73)	47 (26.4)	–	NA	NA
TG	≤ 0.5 to > 1	1	> 1	NA	NA	NA	NA	–	NA	NA
ERV	≤ 0.008 to > 0.5	0.5	> 0.5	NA	NA	NA	NA	–	NA	NA

*AZT/AVI* aztreonam/avibactam, *CAZ/AVI* ceftazidime/avibactam, *MEM/VAB* meropenem/vaborbactam, *IMP/REL* imipenem/relebactam, *MEM* meropenem, *IMP* imipenem, *CFT/TAZ* ceftolozane/tazobactam, *PIP/TAZ* piperacillin/tazobactam, *AZT* aztreonam, *FEP* cefepime, *AK* amikacin, *COL* colistin, *TOB* tobramycin, *TG* tigecycline, *ERV* eravacycline, *NA* not applicable (clinical breakpoints not available)

^a^The concentration range of antibiotic tested was 0.5 to > 4 mg/L. Consequently, only susceptible strains were identified using CLSI breakpoints

Conventional carbapenems (meropenem and imipenem) demonstrated very limited activity, with susceptibility rates below 7%. Among non-β-lactam comparators, colistin exhibited the highest activity (81.2% susceptible by EUCAST and 81.5% intermediate by CLSI; MIC_50_ ≤ 0.5 mg/L; MIC_90_ > 16 mg/L), whereas aminoglycosides such as amikacin and tobramycin showed lower activity rates (32.6 and 27% susceptible by EUCAST, and 21.9 and 26.4% by CLSI, respectively). Tigecycline and eravacycline exhibited MIC_50_ values of 1 mg/L and 0.5 mg/L, and MIC_90_ values of > 1 mg/L and > 0.5 mg/L, respectively (Table 1).

### Phenotypic and genotypic features of KPC-Kp isolates resistant to novel BL/BLICs

A total of 24 KPC-Kp isolates exhibited resistance to at least one of the novel BL/BLICs (Table 2). Four distinct resistance phenotypes were identified: (i) resistance to CAZ/AVI alone, (ii) combined resistance to CAZ/AVI and MEM/VAB, (iii) resistance including IMP/REL, and (iv) resistance to both CAZ/AVI and AZT/AVI.

**Table 2:** Genomic characteristics of KPC-Kp strains resistant to ceftazidime/avibactam and/or aztreonam/avibactam and/or meropenem/vaborbactam and/or imipenem/relebactam

Strain	Year	AZT/AVI (mg/L)	CZA (mg/L)	MEV (mg/L)	IMP/REL (mg/L)	MEM (mg/L)	ST	Serotype	Carbapenemase genes	β-lactamase genes	OmpK35	OmpK36
R6731	2021	0.5	** > 16**	1	0.25	**32**	512	O1/02V2; K107	*bla*_KPC-49_	*bla*_SHV-11_, *bla*_TEM-1_	TR	ins135GD
R6725	2021	0.25	** > 16**	0.5	0.12	0.5	512	O1/02V2; K107	*bla*_KPC-31_	*bla*_SHV-11_, *bla*_TEM-1_	TR	ins135GD
R6729	2021	0.25	** > 16**	1	0.5	1	512	O1/02V2; K107	*bla*_KPC-167_	*bla*_SHV-11_, *bla*_TEM-1_	TR	ins135GD
42t02	2021	**12**	** > 16**	0.25	0.25	2	1685	O1/O2v1; K17	*bla*_KPC-14_	*bla*_TEM_, *bla*_SHV-1_, *bla*_OXA-1_, *bla*_CTX-M-15_	wt	ins136DT
TO-21	2021	1.5	8	** > 16**	2	** > 16**	101	O1/02V1; K17	*bla*_KPC-3_	*bla*_TEM-1_, *bla*_SHV-1_	wt	ins135GD
TO-23	2021	0.5	** > 16**	≤ 0.5	0.25	**16**	101	O1/02V1; K17	*bla*_KPC-2_	*bla*_TEM-1_, *bla*_SHV-1_	wt	ins135GD
TO-24	2021	0.5	** > 16**	1	0.25	2	307	O1/02V2; K102	*bla*_KPC-31_	*bla*_TEM-1_, *bla*_SHV-28,_ *bla*_OXA-1,_ *bla*_CTX-M15_	wt	ins135GD
KPC-KP_TO5	2021	1	8	** > 16**	**8**	** > 32**	512	O1/O2v2; K107	*bla*_KPC-3_	*bla*_TEM1A_, *bla*_OXA-9_	TR	TR
IH-5396	2021	0.5	** > 16**	0.5	0.5	8	512	O1/O2v2; K107	*bla*_KPC-31_	*bla*_KPC-31_, *bla*_OXA-9_, *bla*_SHV-11_, *bla*_TEM-1_	TR	ins135GD
IH-5400	2021	1	** > 16**	1	0.25	4	512	O1/O2v2; K107	*bla*_KPC-121_	*bla*_SHV-11_	TR	ins135GD
IH5397	2022	0.75	** > 16**	0.5	0.12	4	512	O1/O2v2; K107	*bla*_KPC-31_	*bla*_OXA-9_, *bla*_SHV-11_, *bla*_TEM-1_	wt	ins135GD
27ML	2022	0.5	** > 16**	1	0.25	8	512	O1/O2v2; K107	*bla*_KPC-167_	*bla*_TEM-1_, *bla*_SHV-11_	TR	ins135GD
28MF	2022	1	** > 16**	4	0.25	2	512	O1/O2v2; K107	*bla*_KPC-167_	*bla*_TEM-1_, *bla*_SHV-11_	TR	ins135GD
29IP	2022	1	** > 16**	4	0.5	8	101	O1/O2v1; K17	*bla*_KPC-31_	*bla*_SHV-1_, *bla*_CTX-M-15_	wt	ins136DT
29BF	2022	2	** > 16**	8	1	** > 16**	307	O1/O2v2; K102	*bla*_KPC-3_	*bla*_TEM-1_, *bla*_SHV-28_	wt	ins135D
30FC	2022	0.5	**16**	1	0.25	2	512	O1/O2v2; K107	*bla*_KPC-49_	*bla*_TEM-1_, *bla*_SHV-11_	TR	ins135GD
GG03	2022	**8**	** > 16**	2	0.5	4	101	O1/O2v2; K107	*bla*_KPC-14_	*bla*_SHV-1_	wt	ins136DT
TO-17	2022	0.75	** > 16**	0.25	0.5	2	1685	O1/02V2; K107	*bla*_KPC-167_	*bla*_TEM-1_, *bla*_SHV-1_	TR	ins135GD
TO-18	2022	2	** > 16**	**16**	**4**	** > 16**	101	O1/02V1; K17	*bla*_KPC-93_	*bla*_*OXA-1*_, *bla*_SHV-1,_ *bla*_*CTX-M15*_	wt	ins135GD
TO-19	2023	3	** > 16**	4	1	**16**	101	O1/02V1; K17	*bla*_KPC-93_	*bla*_TEM-1_, *bla*_SHV-1_	wt	ins135GD
TO-22	2023	0.38	** > 16**	**16**	** > 8**	** > 16**	512	O1/02V2; K107	*bla*_KPC-3_, *bla*_VIM-1_	*bla*_TEM-1_, *bla*_SHV-11_	TR	ins135GD
RA-02	2023	0.75	** > 16**	1	0.5	4	101	O1/02V1; K17	*bla*_KPC-33_	*bla*_SHV-1_	wt	ins136DT
RA-03	2023	0.5	1	** > 16**	** > 16**	** > 32**	101	O1/02V1; K17	*bla*_KPC-2_	*bla*_TEM-1_, *bla*_SHV-1_	wt	ins136DT
TO-20	2023	0.75	2	**16**	0.5	** > 16**	101	O1/02V1; K17	*bla*_KPC-2_	*bla*_TEM-1_, *bla*_SHV-1_	wt	ins135GD

MIC values falling in the resistance range according to EUCAST breakpoints are indicated with bold character

*TR* truncated, *ins* insertion, *wt* wild type

WGS revealed a genetically heterogeneous population, predominantly belonging to the ST512 (O1/O2v2; K107) and ST101 (O1/O2v1; K17), with a few isolates assigned to ST307 and ST1685. The resistant isolates carried various *bla*_KPC_ alleles co-occurring with additional β-lactamase genes such as *bla*_TEM-1_, *bla*_SHV-11_, *bla*_OXA-1_, and *bla*_CTX-M-15_. Overall, 17 of the 24 isolates resistant to novel BL/BLICs carried mutated *bla*_KPC_ variants (*bla*_KPC-31_, n = 5; *bla*_KPC-167_, n = 4; *bla*_KPC-93_, n = 2; *bla*_KPC-49_, n = 2; *bla*_KPC-14_, n = 2; *bla*_KPC-121_, n = 1; *bla*_KPC-33_, n = 1), while the remaining seven harbored wild-type *bla*_KPC_ alleles (*bla*_KPC-3_, n = 6; *bla*_KPC-2_, n = 1), one of which also carried *bla*_VIM-1_. Nearly all isolates exhibited OmpK36 porin insertions—predominantly ins135GD or ins136DT—while 11 of 24 isolates (45.8%) displayed OmpK35 truncations.

CAZ/AVI resistance (MIC > 8 mg/L) represented the predominant phenotype, detected in 20 of 24 isolates (83.3%), These strains mainly belonged to ST512 (n = 10) and ST101 (n = 6), with addition isolates assigned to ST307 (n = 2) and ST1685 (n = 2). Overall, 17 of the 20 (85%) CAZ/AVI-resistant strains harbored mutated *bla*_KPC_.

MEM/VAB resistance (MIC ≥ 8 mg/L) was observed in six isolates, including four ST101 and two ST512 strains. These carried *bla*_KPC-3_ (n = 2), *bla*_KPC-2_ (n = 2), *bla*_KPC-93_ (n = 1), or *bla*_KPC-3_ plus *bla*_VIM-1_ (n = 1), and all exhibited porin alterations or complete OmpK35 loss. Furthermore, four isolates were co-resistant to IMP/REL, and two of these showed CAZ/AVI resistance.

Resistance to AZT/AVI (MIC > 4 mg/L) was uncommon, identified in two isolates (ST1685 and ST101), both harboring *bla*_KPC-14_ and displaying concomitant CAZ/AVI resistance.

### Clinical characteristics of patients with infections due to KPC-Kp isolates resistant to novel BL/BLICs

The 24 K*. pneumoniae* isolates resistant to at least one novel BL/BLICs were recovered from 23 distinct patients (Table 3). Most patients were male (16/23, 69.6%), with a median age of 59 years (range 21–85). Comorbid conditions were common, including diabetes (5/23, 21.7%), chronic kidney or end-stage renal disease (4/23, 17.4%), cardiovascular disease (4/23, 17.4%), and obesity (3/23, 13.0%). Overall, 20 patients (86.9%) had at least one major comorbidity, and 13 (56.5%) had two or more chronic conditions. Six patients (26.1%) were solid organ transplant recipients, primarily lung or combined liver–kidney transplants.

**Table 3:** Clinical features of patients suffering from KPC-producing *Klebsiella pneumoniae* bloodstream infection included in the study

Patient	Gender, age (years)	Comorbidities	Reason for admission	KPC-Kp isolate	Previous KPC-Kp colonization	Previous novel BL/BLI or 4th-5^th^GC exposure	Targeted antibiotic therapy	Sepsis-associated complications	Timeframe for novel BL/BLI resistance (days)			Length of stay (days)	Outcome
									CZA	MEV or IMR	ATZ/AVI		
1	M, 21	Cystic fibrosis, Diabetes	Lung-liver-pancreas transplantation	R6731	Yes	CZA	CZA + MEM + AK	None	167	–	–	258	Dismissed
2	M, 45	None	Haemorrhagic stroke	R6725	Yes	CZA	AK	None	22	–	–	71	Dismissed
3	M, 53	Congenital encephalopathy	Aspiration pneumonia	R6729	Yes	CZA	CZA + CL	None	27	–	–	192	Dismissed
4	F, 69	Diabetes, Heart failure, CKD	LVAD implantation	42t02	Yes	CZA	MEM	VAP, AKI	5	–	5	148	Dismissed
5	F, 61	Kidney-liver transplantation	Bowel obstruction + pulmonary embolism	TO-21 meV	Yes	CZA	CL + MEM	None	–	170	–	68	Dismissed
6	F, 59	Diabetes	Necrotizing fasciitis	TO-23	Yes	CZA	MEV + AK	None	53	–	–	110	Dismissed
7	M, 40	None	Trauma	TO-24	Yes	CZA	FDC + AK	None	3	–	–	17	Dismissed
8	F, 35	SLE, ESRD and liver cirrhosis	Kidney-liver transplantation	KPC-KP_TO5	Yes	CZA, MEV	MEV + GM	Shock	–	109	–	150	Deceased
9	M, 56	COPD	Lung transplantation	IH-5396	Yes	CZA	MEV + GM	VAP, AKI and shock	88	–	–	122	Deceased
10	M, 59	IHD	Surgery for rectal cancer	IH-5400	Yes	FDC	MEV	None	33	–	–	96	Dismissed
11	M, 82	Diabetes	Burns	IH5397	Yes	FEP, CZA	MEV + FF	None	45	–	–	86	Dismissed
12	F, 59	Obesity, COPD	SARS-CoV-2 pneumonia	27ML	Yes	FEP	MEV + GM	HAP	28	–	–	97	Dismissed
13	M, 77	None	Trauma	28MF	Yes	FEP	MEV + FF	None	13	–	–	30	Dismissed
14	M, 52	None	Burns	29IP	Yes	BPR	MEV + GM	None	30	–	–	57	Dismissed
15	M, 71	Metastatic melanoma	Cachexia	29BF	No	ND	MEV + GM	None	NA	–	–	59	Dismissed
16	M, 70	Obesity, Diabetes	Surgery for rectal cancer	30FC	Yes	CZA	MEV	None	44	–	–	182	Dismissed
17	M, 63	Obesity, IHD, ESRD	Kidney transplantation	GG03	Yes	CZA, FDC	None	Shock, AKI	30	–	30	233	Deceased
18	F, 55	Hypertension	SARS-CoV-2 pneumonia	TO-17	Yes	MEV	MEV + GM	None	30	–	–	162	Dismissed
19	M, 51	Lung transplantation	HAP	TO-18	No	ND	CL + SMX/TMP	HAP	NA	NA	NA	52	Dismissed
20	M, 58	Aortic prosthesis, tracheal stenosis	HAP	TO-19	Yes	ND	MEV + FF	HAP	–	–	–	72	Dismissed
21	M, 62	IHD, tracheal stenosis	HAP	TO-22	Yes	CZA, MEV	MEV + FF	Shock	30	30	–	81	Deceased
22	F, 75	Atrial fibrillation, Aortic bioprosthesis	Heart failure	RA-02	Yes	CZA	IMR	AKI	14	–	–	42	Deceased
				RA-03		IMR	–	–	–	14	–		
23	M, 85	Cerebrovascular disease, heart failure	Sepsis	TO-20	Yes	ND	MEV	None	–	NA	–	25	Dismissed

*KPC-Kp* KPC-producing *Klebsiella pneumoniae*, *BL/BLI* β-lactam/β-lactamase inhibitor, *4th-5*^*th*^*GC* fourth-fifth generation cephalosporin, *CZA* ceftazidime/avibactam, *MEV* meropenem/vaborbactam, *IMR* imipenem/relebactam, *ATZ/AVI* aztreonam/avibactam, *CKD* chronic kidney disease, *LVAD* left ventricular assist device, *MEM* meropenem, *VAP* ventilator-associated pneumonia, *AKI* acute kidney injury, *CL* colistin, *AK* amikacin, *FDC* cefiderocol, *SLE* systemic lupus erythematosus, *ESDR* end-stage renal disease, *GM* gentamicin, *COPD* chronic obstructive pulmonary disease, IHD ischaemic heart disease, *FEP* cefepime, *FF* fosfomycin, *HAP* hospital-acquired pneumonia, *BPR* ceftobiprole, *ND* not documented, *NA* not available, *SMX/TMP* sulfamethoxazole/trimethoprim

The main reasons for admission included post-transplant management, trauma, major abdominal or cardiac surgery, and severe hospital- or ventilator-associated pneumonia (HAP/VAP). Prior intestinal colonization with KPC-Kp was documented in 21 of 23 patients (91.3%), consistent with the known transition from gastrointestinal carriage to invasive infection in high-risk hospitalized hosts.

Prior antimicrobial exposure was universal, as all patients had received at least one course of a novel BL/BLIC or a fourth- or fifth-generation cephalosporin before bloodstream infection onset. The median interval between antimicrobial exposure and detection of the resistant strain in blood culture was 47 days (range 5–167). Previous treatment with CAZ/AVI was documented in 12 of 20 patients (60%) with CAZ/AVI-resistant infections, followed by cefepime (15%) and cefiderocol (10%). Among patients with MEM/VAB- and/or IMP/REL-resistant isolates, half (3/6) had been previously treated with the corresponding agents, suggesting direct selection pressure associated with prior therapy.

Targeted treatment regimens for the 24 BL/BLIC-resistant KPC-Kp bloodstream infections are summarized in Table 3. Combination therapy was used in in 19 cases (79%), while 5 patients (21%) received monotherapy. Novel BL/BLICs were included in 17 treatment courses (71%). MEM/VAB was the most frequently used agent for CAZ/AVI-resistant infections (12/20, 60%), predominantly in combination (9/12) with aminoglycosides (n = 6) or fosfomycin (n = 3). Infections caused by MEM/VAB- or IMP/REL-resistant isolates were primarily managed with non–β-lactam-based regimens, including colistin (2/6) and fosfomycin (2/6). Cefiderocol, combined with amikacin, was used as targeted therapy in one case of CAZ/AVI-resistant bloodstream infection.

Sepsis-associated complications occurred in a substantial proportion of patients, most frequently HAP/VAP, acute kidney injury, and septic shock. Hospitalization was prolonged, with a median duration of 97 days. Five patients (21.7%) – each with severe underlying comorbidities and infections caused by KPC-Kp isolates carrying *bla*_KPC-31_ or *bla*_KPC-93_ died—predominantly due to septic shock and multiple organ failure. The remaining patients achieved both microbiological and clinical cure and were successfully discharged.

## Discussion

This study provides an updated overview of the epidemiology and resistance dynamics of KPC-Kp causing bloodstream infections, with particular emphasis on susceptibility to the newest BL/BLICs and the clinical correlates of resistance. Among the agents tested, AZT/AVI retained the highest in vitro activity (> 99% susceptible), confirming its potent activity against carbapenem-resistant and serine β-lactamase-producing Enterobacterales [7, 23, 24]. MEM/VAB and IMP/REL also demonstrated excellent activity (> 96% susceptible), consistent with previous clinical and surveillance data indicating retained efficacy against *K. pneumonaie* strains harboring both wild-type and mutated KPC [6, 19, 25, 26]. In contrast, CAZ/AVI showed moderate in vitro activity (~ 11% resistant), likely reflecting its earlier and more widespread clinical use relative to other BL/BLICs and the ease with which KPC variants—particularly those with Ω-loop mutations—can emerge to reduce susceptibility [8, 9, 11, 12, 14]. WGS revealed diverse yet convergent mechanisms driving resistance to the novel BL/BLICs, predominantly mediated by KPC enzyme variants and porin defects. Most BL/BLIC-resistant KPC-Kp isolates belonged to high-risk clones ST512 and ST101, in line with previous reports identifying these lineages as primary carriers of *bla*_KPC_ and major contributors to hospital-associated outbreaks [6, 27, 28]. Less frequent detection of ST307 and ST1685 strains indicate ongoing clonal diversification, suggesting that multiple lineages are acquiring resistance mechanisms and contributing to the heterogeneous genetic landscape of KPC-Kp [29, 30].

Resistance to CAZ/AVI was predominant and primarily associated with *bla*_KPC_ variants harboring Ω-loop mutations, which reduce avibactam binding and enhance ceftazidime hydrolysis [13, 31]. These mutations have been increasingly reported in Italy and across Europe, often arising in vivo during CAZ/AVI therapy [12, 14, 32]. The frequent coexistence of *OmpK36* insertions (ins135GD, ins136DT) and *OmpK35* truncations suggests a synergistic contribution of permeability loss, consistent with mechanistic studies demonstrating the combined effect of enzymatic and porin-mediated resistance [11, 33, 34].

Resistance to MEM/VAB and IMP/REL was less frequent and primarily associated with outer-membrane alterations in isolates carrying *bla*_KPC-2_ or *bla*_KPC-3_. This aligns with previous reports indicating that reduced susceptibility to vaborbactam or relebactam is predominantly driven by decreased permeability and increased enzyme expression rather than inhibitor-specific mutations [6, 11, 34]. ATM/AVI resistance was exceedingly rare, observed only in isolates harbouring *bla*_KPC-14_, which also conferred resistance to CAZ/AVI, suggesting a shared structural alteration affecting avibactam binding [35]. Confirmation of this association will, however, require further studies including gene cloning, molecular docking, and molecular dynamics simulations.

Clinically, prior antibiotic exposure represented a major driver in selection of resistant subpopulations. Nearly all patients had received CAZ/AVI or other β-lactams, with a median interval of 47 days preceding the detection of CAZ/AVI resistant KPC-Kp infections—consistent with previous studies demonstrating the rapid emergence of *bla*_KPC_ mutants under avibactam selective pressure [12, 32]. Notably, half of MEM/VAB-resistant infections occurred in patients with prior exposure to the same agent, underscoring that resistance can arise under direct selective pressure even with second-generation inhibitors [11, 12, 33].Therapeutic management of infections caused by BL/BLIC-resistant KPC-Kp were largely individualized and predominantly based on combination regimens. MEM/VAB was most frequently employed for CAZ/AVI-resistant infections, often in combination with aminoglycosides or fosfomycin, whereas infections resistant to multiple BL/BLICs required treatment with non β-lactams-based regimens. Cefiderocol was administered as targeted therapy in a single case of CAZ/AVI-resistant KPC-31-producing *K. pneumoniae* infection. However, its efficacy against KPC variants harbouring Ω-loop mutations remains uncertain, as these mutations have been associated with decreased cefiderocol susceptibility due to enhanced β-lactamase hydrolytic activity [36, 37]. Although evidence supporting the superiority of combination therapy over monotherapy remains limited, such regimens are often justified in critically ill or immunocompromised patients to optimize bacterial clearance and mitigate the risk of therapeutic failure [38, 39].Despite the high prevalence of severe comorbidities and immunocompromising conditions, including solid organ transplantion, sepsis-associated complications were unfrequent and the 28-day mortality rate was 21.7%, notably lower than historical estimates for KPC-Kp bloodstream infections (> 30–40%) [40, 41]. Notably, despite their multidrug-resistant phenotype, some KPC variants did not appear to cause more severe infections or higher mortality rates compared with those producing wild-type KPC enzymes. This may suggest a trade-off between resistance and virulence, reflecting reduced bacterial fitness due to the metabolic cost of maintaining adaptive mutations under selective antibiotic pressure, as reported in previous experimental and clinical studies [42, 43].

This study provides one of the most up-to-date and comprehensive characterizations of resistance to novel BL/BLICs among KPC-Kp bloodstream isolates in Italy, a country with high endemicity. The prospective design and inclusion of consecutive clinical isolates minimize selection bias and enhance representativeness. The integration of WGS with detailed clinical data allowed a robust correlation between genetic resistance mechanisms, antimicrobial exposure, and patient outcomes. However, some limitations should be acknowledged. The study was conducted at a single tertiary centre, which may limit generalizability to other epidemiological contexts or healthcare settings with different antimicrobial use patterns. The sample size of resistant isolates was relatively small, constraining statistical power for identifying independent clinical predictors of resistance or mortality. Additionally, the focus on bloodstream infections may not capture the full diversity of resistance mechanisms present in other infection sites or colonizing strains. Pharmacokinetic and pharmacodynamic parameters were not evaluated, precluding assessment of potential subtherapeutic exposures contributing to resistance selection. Finally, while WGS provided comprehensive genotypic data, functional validation of novel mutations was not performed, limiting mechanistic inference.

In conclusion, our findings highlight the ongoing evolution of resistance mechanisms among KPC-Kp in the era of novel BL/BLICs. While MEM/VAB, IMP/REL, and AZT/AVI continue to exhibit high efficacy, resistance to CAZ/AVI is increasingly reported, primarily driven by mutations in the *bla*_KPC_ gene. Sustained genomic surveillance, judicious antimicrobial use, and the implementation of optimized therapeutic strategies are essential to preserve the clinical utility of these last-resort agents and to curb the dissemination of high-risk resistant clones.

## Data Availability

The authors confirm that the data supporting the findings of this study are available upon reasonable request.
